# Effects of Community-Based Physical-Cognitive Training, Health Education, and Reablement among Rural Community-Dwelling Older Adults with Mobility Deficits

**DOI:** 10.3390/ijerph18179374

**Published:** 2021-09-05

**Authors:** Chen-Yi Song, Pay-Shin Lin, Pei-Lun Hung, ADLers Occupational Therapy Clinic

**Affiliations:** 1 Department of Long-Term Care, National Taipei University of Nursing and Health Sciences, Taipei 112303, Taiwan; ax911142@gmail.com; 2 Department of Physical Therapy, Graduate Institute of Rehabilitation Science, College of Medicine, Chang Gung University, Taoyuan 33302, Taiwan; pslin@mail.cgu.edu.tw; 3 Master Degree Program in Healthcare Industry, College of Medicine, Chang Gung University, Taoyuan 33302, Taiwan; 4 Healthy Aging Research Center, Chang Gung University, Chang Gung Memorial Hospital, Taoyuan 33302, Taiwan; 5 ADLers Occupational Therapy Clinic, Taipei 10491, Taiwan; adlers0615@gmail.com

**Keywords:** restorative care, reablement, function, elderly, long-term care

## Abstract

Reablement services are approaches for maintaining and improving the functional independence of older adults. Previous reablement studies were conducted in a home environment. Due to the limited evidence on the effects of multicomponent interventions and reablement in a community-based context, this study aimed to develop and evaluate the effect of community-based physical–cognitive training, health education, and reablement (PCHER) among rural community-dwelling older adults with mobility deficits. The trial was conducted in rural areas of New Taipei City, Taiwan. Older adults with mild to moderate mobility deficits were recruited from six adult daycare centers, and a cluster assignment was applied in a counterbalanced order. The experimental group (*n* = 16) received a PCHER intervention, comprising 1.5 h of group courses and 1 h of individualized reablement training, while the control group (*n* = 12) underwent PCHE intervention, comprising 1.5 h of group courses and 1 h of placebo treatment. A 2.5-h training session was completed weekly for 10 weeks. The outcome measures contained the de Morton Mobility Index (DEMMI), the Saint Louis University Mental Status (SLUMS) Examination, the Barthel Index (BI), the Short Physical Performance Battery (SPPB), and the Canadian Occupational Performance Measure (COPM). The PCHER significantly improved the DEMMI, SLUMS, BI, SPPB, and COPM (all *p* < 0.05), with medium-to-large effect sizes. PCHER also showed an advantage over PCHE in terms of the SPPB (*p* = 0.02). This study verified that combining individualized reablement with group-based multicomponent training was superior to group courses alone in enhancing the functional abilities of community-dwelling older adults with mobility deficits.

## 1. Introduction

Reablement services are approaches for maintaining and improving the functional independence of older adults [1]. The intervention is targeted, focused on enhancing the performance of daily activities defined as important by the person, and takes place in the person’s home and local surroundings [2]. The aim is to enable people to age in place, be active and participate socially and in society [2]. Previous studies demonstrated some positive impacts of reablement on functional abilities, health-related quality of life, and reduction in healthcare service utilization [3,4,5]. For older adults with mild disability, reablement was found to enhance independence from long-term care services [6]. However, most reablement studies were conducted in the context of home care [1,2,3,4,5]. Community-dwelling older people with physical dysfunction may use other care services, such as adult day care. There is limited knowledge regarding reablement in a community-based context.

A one-year follow-up study reported that limitations in lower limb function is a risk factor for functional decline among older adults in rural areas [7]. As lower extremity function may predict future disability [8], improving functional mobility deficits to prevent further disability progression is vital. Physical exercise has been proposed for improving physical performance in the community-dwelling elderly, including those in daycare centers [9,10,11]. On the other hand, rural areas in Taiwan have relatively low financial resources and less access to multiple services. Older adults in rural communities have various health problems, including sarcopenia, cognitive impairment, malnourishment, and a susceptibility to falls [12,13,14,15].

Multicomponent interventions are generally more effective than single-component interventions. Combined physical and cognitive training, either simultaneous or subsequent, is more successful relative to physical or cognitive exercises alone [16]. Reablement programs combined with standard care (e.g., long-term care services such as home help or day care services) were found to be superior to standard care alone in enhancing the independence of older adults [6]. In Cho et al. [17], a multicomponent intervention program, comprising an exercise component (focusing on balance and muscle strength) and a fall education component, reduced the fall risk for community-dwelling older adults. The aforementioned studies implied that combining physical–cognitive training, health education, and reablement (PCHER) is beneficial to rural community-dwelling older adults.

The purpose of the present study was to develop and evaluate the effect of a community-based intervention combining PCHER among rural community-dwelling older adults with mobility deficits compared with a control in relation to mobility, cognitive function, activities of daily living (ADL) function, physical function, and self-perceived activity performance and satisfaction with performance.

## 2. Materials and Methods

### 2.1. Study Design and Participants

This is a controlled before-and-after study [18] conducted from September to November 2019 in six rural areas of New Taipei City, Taiwan. Older adults were recruited from six public adult daycare centers. The participants of each adult daycare center were assigned in a counterbalanced order into the experimental and control groups. Each group comprised older adults from three adult daycare centers. The study protocol was approved by the Institutional Review Board of the Antai-Tian-Sheng Memorial Hospital, and the trial was registered in the ClinicalTrials.gov (NCT04311138).

The inclusion criteria were (1) aged 65 years or older; (2) had mild to moderate mobility deficits (de Morton Mobility Index (DEMMI) score 39–67 [19,20]); and (3) had a gait speed of ≤1 m/s [21]. The exclusion criteria included the following: had moderate to severe cognitive impairment or a Clinical Dementia Rating score of ≥2 [22]. All participants provided signed informed consent prior to participating.

### 2.2. Intervention

#### 2.2.1. PCHER

The PCHER program is a community-based intervention program. Adult daycare centers for the experimental group were visited by two rehabilitation specialists (i.e., qualified physiotherapist or occupational therapist) weekly for intervention. Each intervention session lasted 2.5 h, comprising 1.5 h of group courses and 1 h of individualized reablement training. The intervention spanned 10 weeks

The group training course covered physical training, cognitive training, and health education, which was designed according to problems frequently encountered by older adults in their daily lives. Training was led by a therapist using presentation slides. Both knowledge and skill were taught prior to practice (Table 1). After the group course ended, therapists provided one-on-one reablement training to each participant. At the initial visit assessment, a care goal was formulated after discussion between participants and therapists to identify the problems they encountered and perceived as the most important in performing self-care, productive activities, and leisure activities.

#### 2.2.2. Physical–Cognitive Training and Health Education (PCHE)

The control group also received 1.5 h of group courses (Table 1), but, in place of individualized reablement intervention, they were given placebo treatments, including therapists to accompany and chat with them in sitting position.

### 2.3. Outcomes

The main outcome measurements included mobility, cognitive function, ADL function, physical function, and self-perceived activity performance and satisfaction with performance. Assessments were performed before and after the interventions by a blinded examiner.

Mobility function was measured using the DEMMI. The DEMMI is a 15-item unidimensional instrument that measures mobility across the spectrum from bed bound to independent mobility and has been rigorously developed and validated [19]. The Rasch converted interval DEMMI score ranges from 0 to 100, with higher scores indicating better mobility. A DEMMI score of 20–36, 39–67, and 74–100 signify severe, mild to moderate, and no mobility difficulty, respectively [20].

Cognitive function was assessed using the Saint Louis University Mental Status (SLUMS) Examination, which was validated using a sample of community-dwelling older adults [24]. The SLUMS is a 30-point questionnaire that tests for orientation, memory, attention, and executive functions. The mild cognitive impairment (MCI) cutoff scores for individuals with at least high school education and less than high school education were 21–26 and 20–24, respectively. The dementia cutoff scores for individuals with at least high school education and less than high school education were <21 and <20, respectively [25]. In this study, we used SLUMS-Chinese version [26]; the scores to detect dementia for Chinese population with at least high school education and less than high school education were <24 and <22, respectively [27].

The Barthel Index (BI) is a reliable and valid tool for measuring ADL function [28]. It contains the following 10 items: feeding, grooming, dressing, bathing, bowel control, bladder control, toileting, transferring, ambulation, and stairs climbing. The total score ranges from 0 to 100; a score of 0–20 indicates “total” dependency, 21–60 indicates “severe” dependency, 61–90 indicates “moderate” dependency, and 91–99 indicates “slight” dependency.

The Short Physical Performance Battery (SPPB), a valid tool, was applied to assess physical performance, including standing balance, walking, and five times sit-to-stand [8]. For each test, the time required was recorded and converted into points (0–4), thereby giving a total score of 0–12 points, with higher scores representing better physical function. A total score ≤9 points can distinguish frail from non-frail individuals [29] and is used as low physical performance criteria for sarcopenia [30].

The Canadian Occupational Performance Measure (COPM), with adequate psychometric properties, was used to measure participants’ self-perceived activity performance (COPM_P) and satisfaction with performance (COPM_S) within the following 3 areas: self-care, productivity, and leisure [31]. During a semi-structured interview, participants were asked to prioritize five of the most important activities and thereafter rate the level of performance and level of satisfaction on a scale from 1 to 10. A higher score reflects better performance and higher satisfaction.

### 2.4. Statistical Analyses

Statistical analysis was performed using SPSS 23.0 (IBM, Armonk, NY, USA). Shapiro–Wilk test was employed to test for data normality. The Chi-square test and Mann–Whitney *U* test were conducted to determine differences between groups with respect to baseline characteristics of the participants and pre-intervention scores. The Wilcoxon signed-rank test was subsequently employed for comparison outcome variables of interest between pre- and post-assessments for each group, and the Mann–Whitney *U* test was used to examine the differences in score changes between groups. The statistical significance was set at α = 0.05. Effect sizes for nonparametric data were calculated with the formula *r* = *Z*/√*n*, and classified as small (*r* = 0.1), medium (*r* = 0.3), and large (*r* = 0.5) [32].

## 3. Results

### 3.1. Participants

Twenty-eight elders were enrolled in the study from six adult daycare centers. The PCHER group and the PCHE group enrolled 16 elders (six, six, and four from the three centers) and 12 elders (seven, two, and three from the three centers), respectively; the response rate ranges from 20 to 47%. All of them completed the study. The demographic information for the study participants is presented in Table 2. The two groups did not exhibit significant differences except education attainment and illness. The participants in the PCHER group had higher education and a higher percentage of diabetes.

### 3.2. Outcomes

The pre-intervention scores were similar between the PCHER and PCHE groups except for the SPPB (*p* = 0.01), walking (*p* = 0.24), and five times sit-to-stand (*p* = 0.01) scores (Table 3). No statistically significant differences were found in the comparison of post-intervention scores between the two groups. However, the PCHER group exhibited significant post-intervention improvements in the DEMMI, SLUMS, BI, SPPB, and COPM_S scores (all *p* < 0.05, Table 3). In terms of the COPM_P, the improvement was marginally significant (*p* = 0.055), with a medium effect size (*r* = 0.48). The PCHE group only demonstrated significant improvements for the DEMMI and COPM_S scores. The PCHER group also demonstrated greater improvements in the SPPB scores than the PCHE group did (*p* = 0.02). In terms of effect size, the PCHER group demonstrated greater improvements on the DEMMI, SLUMS, BI, SPPB, and COPM_P scores when compared with those for the PCHE group.

## 4. Discussion

The present study found that combining individualized reablement and group-based physical–cognitive training and health education had greater effects on lower extremity function, mobility, cognitive function, and ADL function, relative to a control, among rural community-dwelling older adults with mild to moderate mobility deficits. Our findings added knowledge to reablement in a community-based context. As functional abilities are essential indicators of reablement [1,3,5], our findings support the potential use of reablement in the context of community for improving the mobility and functional independence of community-dwelling older adults.

This reablement study considered older adults with mild to moderate mobility deficits. The BI assessment (>90) indicated they were mildly dependent. However, the DEMMI and SPPB scores indicated that functional mobility decline or deficits influenced their physical performance. The evidence suggests that physical exercise interventions could improve the performance-based measures of physical function (e.g., gait speed and SPPB scores) in community-dwelling, frail older adults [9,33]. The combined center- and home-based multicomponent exercise programs also improved physical performance in the older adults [34]. Additionally, research on home-based reablement revealed an improvement of the SPPB after a period of reablement service [35,36]. The current study findings corroborate those of previous studies indicating a combination of group exercise and individualized reablement effectively improved mobility and physical function in older adults, thereby improving their everyday functionality. The positive result observed by this study was related to the multicomponent lower extremity training provided in the PCHER program. However, the significant changes of the SPPB, walking, and the five times sit-to-stand scores in the PCHER group should be interpreted with caution because the lower baseline scores of this group made the room for improvement larger.

The items represented in the SPPB were markers for identifying preclinical disability in community-dwelling older adults [37]. A change of 1.0 point for the SPPB and 0.10 m/s for gait speed were considered substantial [38]. The score changes of the SPPB (1.9-point) in our study were higher than those of reablement studies [35,36]. After the PCHER, time taken for 4-m walking test significantly improved from 10.9 ± 7.5 to 7.7 ± 3.8 s (gait speed: from 0.5 ± 0.2 to 0.6 ± 0.2 m/s); the time taken to complete five sit-to-stand significantly improved from 24.5 ± 14.1 to 18.1 ± 15.0 s. Despite the significant improvement of the SPPB, nine (75%) participants in the PCHER group had a post-intervention score ≤ 9, suggesting a frail or sarcopenia risk [29,30]. Exercise twice a week and a contribution of 1.0, 1.2 to 1.5 g/kg of protein/day and 3 g of beta-hydroxy-beta-methylbutyrate (HMB, leucine precursor) per day with a supplementation period of 8 to 12 weeks enhanced leg muscle mass and knee extensor strength [39]. Future studies could increase the exercise frequency or nutritional supplementation to improve the muscle mass, strength, and function in older adults.

The participants with mild to moderate mobility difficulty in the present study had a DEMMI score of 41–67. The finding was in line with those of a previous study that found that community-dwelling older adults who ambulated with an aid had a DEMMI score of 64.1 ± 12.4 [40]. A Taiwan study showed the elderly with a DEMMI score of 39–67 frequently had disabled ADL items of climbing and ambulation [20]. Additionally, DEMMI scores <48 and <67 could predict an inability to ambulate close to the participant’s residence and to use public transportation, respectively [41]. Community ambulation are crucial for older adults to leave their homes and mingle with their community. After PCHER intervention, the mean DEMMI score exhibited a significant 15.3-point increase. The improvement is clinically meaningful and relevant because a score change of 13 points or larger could be considered significant and clinically important in the community setting [40].

In addition to the improvement of functional abilities using objective measures, the PCHER group also showed subjective improvements in the COPM_P and the COPM_S with medium-to-large effect sizes. Interestingly, the PCHE group also reported increased levels of the COPM_S. A similar phenomenon has been reported in previous studies [2,36]. The authors suggest that the improvement may be caused by the therapeutic effect of the baseline COPM interview, which increases the control group’s awareness of their activity limitations and prompts them to seek solutions themselves [2]. Another possible explanation is the participants in the PCHE group received companionship from the therapists.

Cognitive training can be effective in improving various aspects of objective cognitive functioning, including memory performance, executive functioning, processing speed, attention, fluid intelligence, and subjective cognitive performance [42]. In the current study, the cognitive functions of older adults significantly improved in the PCHER group but not in the PCHE group, indicating that a 1.5-h group course, comprising a cognitive training component, could only maintain cognitive function. We could not eliminate the possibility that the fact that cognitive functions did not improve in the PCHE group may be due to the lower educational level of this group. The possible benefit of an addition of an individualized reablement component on cognition may be associated with physical activity or exercise, which are common strategies integrated in the reablement intervention [43]. The studies on older subjects with MCI reported some positive effects of physical exercise on cognition, mainly on global cognition, executive function, attention, and delayed recall [44].

Lower mean years of education (<6) and poor cognitive function were reported for rural-dwelling older adults [12]. Most of our participants were illiterate (35.7%) or had an elementary school level of education (32.1%). We noticed that they had a low SLUMS score of 14–19. For individuals with an education level lower than high school, the SLUMS scores <20 [26] or 22 [27] indicate dementia. However, relevant studies have not focused on older adults with extremely low education levels. Nevertheless, because of impaired cognitive function, intervention targeting mental health is recommended for rural-dwelling older adults.

The present study had some limitations. First, the trial was not randomized, and the sample size was small, which limits the generalizability of our findings. A sample size of 12 per group met the rule of thumb for a pilot study [45]. Second, the intervention frequency was only once weekly because the therapists were required to travel to six rural adult daycare centers to perform the interventions. Third, only older adults with mild to moderate mobility deficits were recruited because individualized reablement training was provided by two therapists. The dilemmas, however, are faced while delivering reablement services to a remote area. Finally, we did not conduct follow-up measurements. Taken together, future studies may utilize a high-quality design, enroll more participants, and integrate more resources to increase the intervention frequency or duration to accumulate evidence-based information that can inform best practices.

## 5. Conclusions

This study verified that combining individualized reablement with group-based multicomponent training was superior to group courses alone in enhancing the functional abilities of community-dwelling older adults with mobility deficits. Future studies with follow-up measurements are warranted.

## Figures and Tables

**Table 1:** Group-based physical–cognitive training and health education.

Week	Content
1st	Sit-to-stand exercise (10 repetitions/set, 10 sets) Cognicise [23] (e.g., marching with simultaneously clapping, subtracting or adding numbers)
2nd	Static stretching exercises: Neck, shoulder, forearm, spine, gluteus, thigh and calf (10 min/set) Cognitive finger exercises with various gestures
3rd	Pelvic floor exercises (5 s contraction and 20 s resting, 10 repetitions/set) Health education: Improving sleep quality
4th	Fall prevention and balance exercise: Squat, heel raises, and lunge (each 10 repetitions/set) Health education: Antidementia diets
5th	Exercise: How to get back up after a fall (3 repetitions) Health education: Long-term care resources and assistive device application
6th	Squatting exercise (10 repetitions/set, 10 sets) Health education: Oral exercise
7th	Spinal decompression and stretching exercise (20 min) Visual processing exercise (e.g., hands up)
8th	Cognitive games and brain exercises: Visual attention, visual spatial and closure, and visual discrimination Abdominal breathing exercise (5 times/minute, 5 repetitions/set)
9th	Aerobic exercise: Walking (20 min) Health education: Medication management
10th	Antidementia: The five senses

**Table 2:** Demographic information and basic characteristics of the participants.

Characteristics	PCHER (*n* = 16)	PCHE (*n* = 12)	*p*-Value
Gender			0.227
Male	6 (37.5)	2 (16.7)	
Female	10 (62.5)	10 (83.3)	
Age	79 (65–99)	82 (69–91)	0.318
Body height	157.5 (145–170)	155.5 (145–167)	0.415
Body weight	61.9 (40–86)	53.0 (38–80)	0.150
Education			0.030 *
Elementary school and below	10 (62.5)	12 (100)	
Middle school	3 (18.8)	0	
High school	2 (12.5)	0	
College and above	1 (6.2)	0	
Solitary	2 (12.5)	1 (8.3)	0.724
Illness	1 (0–3)	0.5 (0–1)	0.050
Cardiovascular diseases	4 (25.0)	0	0.061
Hypertension	9 (56.3)	6 (50.0)	0.743
Diabetes	5 (31.3)	0	0.033 *

PCHER = physical–cognitive training, health education, and reablement, PCHE = physical–cognitive training and health education. Data were presented as median (range) or number of cases (percentages). * *p* < 0.05.

**Table 3:** Comparisons of mobility, cognitive function, ADL function, physical function, and self-perceived activity performance and satisfaction with performance.

Outcome	PCHER (*n* = 16)				PCHE (*n* = 12)			
	Pre-Intervention	Post-Intervention	*p*	Effect Size	Pre-Intervention	Post-Intervention	*p*-Value	Effect Size
DEMMI	57 (41–67)	67 (44–100)	0.007 *	0.68	67 (53–67)	67 (48–100)	0.046 *	0.58
SLUMS	19 (3–27)	21.5 (3–30)	0.028 *	0.55	14 (6–27)	17 (3–30)	0.610	0.15
BI	95 (85–100)	100 (85–100)	0.026 *	0.56	97.5 (90–100)	100 (85–100)	0.414	0.24
SPPB	6 (2–9)	8 (3–12)	0.005 *	0.70	9 (5–12)	9 (5–12)	0.915	0.03
Balance	3.5 (0–4)	4 (1–4)	0.161	0.35	4 (2–4)	4 (2–4)	0.317	0.29
Walking	2 (1–3)	2 (1–4)	0.010 *	0.65	2.5 (1–4)	2 (1–4)	1.000	0
Five times sit-to-stand	1 (0–3)	2 (0–4)	0.027 *	0.55	3 (1–4)	2.5 (1–4)	0.593	0.15
COPM								
Performance	4.5 (1–10)	5 (2–10)	0.055	0.48	5 (3–10)	6 (3–10)	0.223	0.35
Satisfaction	5 (3–10)	5.5 (3–10)	0.048 *	0.49	5 (1–10)	6 (3–10)	0.017 *	0.69

DEMMI = de Morton Mobility Index, SLUMS = Saint Louis University Mental Status Examination, BI = Barthel index, SPPB = Short Physical Performance Battery, COPM = Canadian Occupational Performance Measure. Data were presented as median (range). * Significant differences between pre- and post-intervention (*p* < 0.05).

## Data Availability

The datasets used and/or analyzed during the current study are available from the corresponding authors on reasonable request.
